# Rapid, responsive, relevant (R3) research: a call for a rapid learning health research enterprise

**DOI:** 10.1186/2001-1326-2-10

**Published:** 2013-05-10

**Authors:** William T Riley, Russell E Glasgow, Lynn Etheredge, Amy P Abernethy

**Affiliations:** 1 National Cancer Institute, Bethesda, MD, USA; 2 George Washington University, Washington, DC, USA; 3 Duke University School of Medicine, Durham, NC, USA

**Keywords:** Research methods, Rapid learning, Research review, Stakeholder engagement, Rapid research, Research relevance, Dissemination and implementation

## Abstract

Our current health research enterprise is painstakingly slow and cumbersome, and its results seldom translate into practice. The slow pace of health research contributes to findings that are less relevant and potentially even obsolete. To produce more rapid, responsive, and relevant research, we propose approaches that increase relevance via greater stakeholder involvement, speed research via innovative designs, streamline review processes, and create and/or better leverage research infrastructure. Broad stakeholder input integrated throughout the research process can both increase relevance and facilitate study procedures. More flexible and rapid research designs should be considered before defaulting to the traditional two-arm randomized controlled trial (RCT), but even traditional RCTs can be designed for more rapid findings. Review processes for grant applications, IRB protocols, and manuscript submissions can be better streamlined to minimize delays. Research infrastructures such as rapid learning systems and other health information technologies can be leveraged to rapidly evaluate new and existing treatments, and alleviate the extensive recruitment delays common in traditional research. These and other approaches are feasible but require a culture shift among the research community to value not only methodological rigor, but also the pace and relevance of research.

## Background

**Figure 1:**
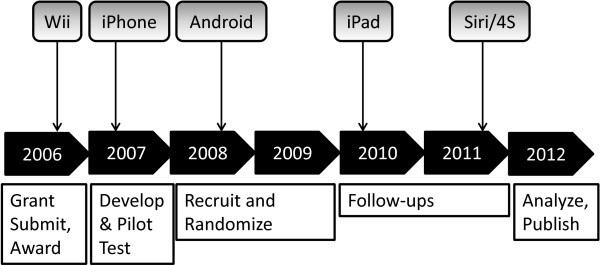
Consumer technology advances missed during a typical RCT published in 2012.

Despite increasing demands to produce timely and relevant research findings, our traditional research process remains painstakingly slow. Randomized efficacy trials take approximately 5.5 years from the initiation of enrollment to publication [1], and 7 years or longer after adding the time from grant application submission to enrollment initiation. Extensive follow-up periods for relevant outcomes such as morbidity/mortality as well as delays in participant recruitment and publication can extend this time period to a decade or longer. During this period, scientific and technological advances will occur that may make the eventual findings less relevant or even obsolete. For illustration, Figure 1 shows a few of the salient consumer technologies introduced during a typical seven year clinical trial. These recent advances in consumer technologies are most impactful for mobile and wireless health research, [2] but many less mainstream scientific and medical technological advances also occur while clinical trials are being conducted. For example, one explanation for the recently reported negative results of the SAMMPRIS trial was that stent technologies and surgery procedures had advanced substantially since study initiation [3,4].

This protracted period from concept to publication is further exacerbated by the slow and limited uptake of research findings into practice. Balas and Boren have estimated that it takes approximately 17 years from concept to evidence implementation for the 14% of evidence that progresses to implementation [5]. A recent report from the President’s Council of Advisors on Science and Technology (PCAST) estimated that 3,000 treatments are in development and concluded that major upgrades are needed in the research system to evaluate these treatments [6]. An Institute of Medicine report on clinical trials states that “recognition is growing that the clinical trials enterprise in the United States faces substantial challenges impeding the efficient and effective conduct of clinical research to support the development of new medicines and evaluate existing therapies [7].” Clearly, our current research enterprise is too slow, inefficient, and cumbersome to meet the rapidly evolving demand.

What are needed are “rapid-learning research systems” that integrate researchers, funders, health systems, practitioners, and community partners asking clinically relevant questions, using efficient and innovative research designs, and leveraging rich, longitudinal data sets from millions of patients. To begin progress toward such a system, we considered and have described approaches to make research more rapid, responsive and relevant (R3), organized into four sections for the purpose of this paper: 1) stakeholder engagement, 2) design, 3) review, and 4) infrastructure (see Table 1). These suggested approaches are viewed as a starting point for a dialogue among the health research community to challenge our current cumbersome research enterprise and to consider these or other approaches to maintain scientific rigor while speeding the process by which more responsive and relevant research findings are produced.

**Table 1:** Issues in promoting rapid research by stage of research

	**Concept through trial preparation**	**Recruitment through follow-up**	**Analysis through publication**
Stakeholder Relevance	&z.cirf; Engage stakeholders via evaluability assessment to assist with design of practical trials &z.cirf; Consider outcomes and measures important and relevant to stakeholders who will need to act on results &z.cirf; Establish stakeholder “citizen-scientist” feedback panels; leverage networking technologies.	&z.cirf; Ongoing engagement with stakeholders on methods to improve recruitment and follow-up retention &z.cirf; Submit preliminary findings to stakeholders for review and direction-setting	&z.cirf; Submit initial results to stakeholders for assistance with interpretation, relevance, dissemination and forming next study questions &z.cirf; Share presentations with stakeholders at policy and practice venues
Design Issues	&z.cirf; Replace the traditional pilot with iterative N-of-1 and optimization designs	&z.cirf; Consider within-subject and MINC to typical comparison conditions &z.cirf; Leverage technology to automate RCTs when possible &z.cirf; Consider alternatives to the two-arm RCT including factorial, within subject, pragmatic, quasi-experimental, and rapid learning designs	&z.cirf; Report proximal outcomes while follow-up data collection continues
Review Issues	&z.cirf; Streamlined grant review process &z.cirf; Encourage reviewers to consider innovative designs that speed research &z.cirf; Streamline IRB approval process, especially for low risk studies	&z.cirf; Rapid modification approvals from IRBs	&z.cirf; Encourage online and open access publication &z.cirf; Incentives to speed manuscript reviews
Infrastructure Issues	&z.cirf; Use of data standards and common data elements to improve research efficiency and facilitate data sharing &z.cirf; Create rapid learning systems that can generate data to test multiple competing hypotheses and develop predictive models &z.cirf; Create national biobank/bio-samples systems	&z.cirf; Use practice network registries to speed recruitment, provide enriched histories & follow-ons &z.cirf; Leverage existing EHR and other rapid learning data systems to rapidly test hypotheses	&z.cirf; Robust policies and procedures for data sharing and merging &z.cirf; Improved systems for disseminating findings to appropriate stakeholders

### Relevance and stakeholder engagement

Broad stakeholder engagement involving patients, providers, health plans, policy makers and other relevant stakeholders may seem counterintuitive as a strategy to speed research, but this time investment has the potential to improve the recruitment and retention of study participants, thus increasing the pace of conducting the study. More importantly, stakeholder engagement increases the likelihood that findings will be relevant to stakeholders and more readily adopted into practice, thereby making the overall research pipeline more efficient. These “evaluability assessments” [8] or participatory approaches are considered key to facilitating the adoption of research findings by practitioners [9]. The Patient Centered Outcomes Research Institute (PCORI), for example, is creating public advisory groups and soliciting patient input on specific comparative effectiveness questions that are relevant to practitioners and stakeholders [10].

An ongoing relationship between researchers, healthcare providers, health plans, and patients is critical to a better, faster research system. Clinical trial recruitment is a major problem with about 90% of US trials failing to meet enrollment goals [7]. The NIH Health Care System Collaboratory (HCSC) offers an important resource for rapid research. Like the HMO Research Network and the VA QUERI program, the HCSC will make available opportunities to conduct large-scale studies within well-organized healthcare delivery systems [11]. Research embedded in organized delivery systems and networks enhances not only research relevance, but also facilitates recruitment, retention, study start-up, operations, data capture, and integration into practice.

An accelerated research system can also use information technologies to speed the process of seeking and obtaining stakeholder feedback. Consistent with a citizen-scientist model [12], a virtual network of various stakeholders can participate and provide feedback throughout the research process using online surveys, virtual meetings, and social media systems [13]. Via innovative technologies, stakeholder feedback can be obtained efficiently to increase research relevance and responsiveness, even for researchers who are not fully integrated into the practice setting or community where their research findings are likely to be translated into practice.

### Rapid research designs

Traditional study designs and procedures are well-established, rigorous, and notoriously slow and costly. This belabored research process typically begins with pilot trials that we posit have limited benefit, are used inappropriately to estimate effect size [14], and often prematurely concretize a less than optimal intervention. Instead, we recommend replacing the traditional pilot trial with a more flexible iterative intervention testing and optimization approach, analogous to the agile software development process that places a premium on failing early to succeed later [15]. For example, N-of-1 trial designs provide intervention development flexibility. With the increasing availability of intensive longitudinal data from wireless sensors and mobile devices, N-of-1 trials can be rapidly implemented and provide results congruent with a more personalized medicine approach [16], and Bayesian analyses from a series of such trials [17] may provide sufficient evidence of generalizability to limit the need for a larger trial.

Intervention optimization designs such as fractional factorial and sequential multiple assignment research trials (SMART) are particularly valuable when the intervention development questions involve combinations or sequences of intervention components [18]. Dynamic system models have also been used to optimize treatments [19]. Some optimization approaches may take more time than the traditional pilot trial, but the pace of the overall research enterprise will be improved by more quickly discarding or modifying interventions that are unlikely to be found effective in larger and more expensive trials.

Within the traditional RCT, researchers have a number of design decisions that can increase efficiency. Trials that utilize within-group designs in which participants serve as their own controls can speed the research process by reducing the number of study participants needed to detect outcomes, and can often simplify study procedures as well. The Minimal Intervention Needed for Change (MINC) standard [20] provides a standard pragmatic comparison anchor across studies for comparative effectiveness research. The VA is adopting a “point of care” randomization that computer-randomizes patients to different treatments, and then uses adaptive algorithms to change allocation of new patients as evidence accumulates [21]. Recent technological advances make it possible to conduct “automated RCTs” in which the enrollment, random assignment, intervention delivery, and outcome assessments are fully automated. To fully realize the potential of automated RCTs and other rapid learning systems, the nature of and procedures for informed consent need to be resolved.

Follow-up periods also can be shortened or segmented. Results can be analyzed at the point where the maximal benefit of the intervention is hypothesized to occur. Longer-term outcomes can be modeled from these results, or one of the investigators can remain blind to conduct the follow-up portion of the study and publish the follow-up results separately.

In addition to improving the efficiency of RCTs, we also need to consider alternative designs that may be more appropriate to the research question and provide more rapid and relevant answers. A range of within-subject and quasi-experimental designs such as interrupted time series [22], stepped wedge [23], and regression discontinuity [24] may have less internal validity than the RCT, but offer a number of advantages [25]. For example, these quasi-experimental approaches facilitated participation of the major Minnesota health insurers in the DIAMOND depression treatment program [26]. These designs may be particularly appropriate for evaluating treatments already adopted in practice.

### Rapid review processes

It takes 9 to 11 months from NIH grant submission to funding [27]. If revised and resubmitted, and assuming a six month revision period, it can take two years from initial submission to the award of a revised (A1) NIH grant application. During this time, science and technology continue to advance; research partnerships, especially with non-research stakeholders, must be maintained; and the research questions may become less relevant or timely.

Grant review and funding processes could be streamlined in a number of ways. For the Recovery Act Challenge Grants [28], a flexible, two-stage review process was implemented to reduce to five months the time from receipt to funding. Rapid review processes are already used by the NIH for time sensitive natural experiments [29,30]. In response to the SARS (Severe Acute Respiratory Syndrome) outbreak, the Canadian Institutes of Health Research developed and issued a funding announcement that resulted in 18 submissions within 2 weeks, and these submissions were reviewed and four approved for funding within 10 days of submission [31]. These rapid review examples clearly indicate that it is possible to review and fund research applications quickly when necessary, and that such rapid review systems should be considered for a broader range of research, including timely and pressing clinical and public health questions.

The grant application review process could facilitate more rapid research not only by reviewing more efficiently, but also by placing a greater premium on more rapid and innovative research designs. Despite the addition of innovation as an NIH review criteria, a recent study of grant applications revealed that novelty is associated with a 4.5 percentile point drop, and that feasibility concerns did not contribute substantially to this “novelty penalty” [32]. Innovative designs, especially those that speed the pace of research relative to traditional designs, should be rewarded, not penalized.

Institutional Review Boards (IRBs) also should consider streamlining review procedures. Slowness of research should be considered a risk, both to study participants who may continue in their assigned treatment even as newer treatments become available, and to the broader public who are delayed getting answers to relevant research questions. Revisions to the Common Rule are anticipated to allow for a more flexible and rapid review process [33].

Online and open access publication practices have greatly reduced the time from acceptance to publication [34] but could be further facilitated by a better or more incentivized process for acquiring reviewers and obtaining reviews. As Green noted, new technologies for publication, systematic reviews, and dissemination of evidence-based guidelines reduce the time from research findings to practitioner adoption, but the publication and dissemination process should continue to be reviewed to further reduce the time lapses between the various stages of the dissemination and implementation process [9].

### Infrastructure for rapid research

Improving our research infrastructure has the potential not only to speed the pace of research, but also increase its rigor and relevance. The health system has lagged decades behind other sectors in IT implementation [35]. As a result, health research has been severely constrained by a data-poor environment in which acquiring needed research data is expensive, difficult, and time-consuming. Since the rapid-learning health system and learning healthcare system concepts were advanced in 2007 [36], major investments have been made in databases and learning networks to take advantage of the research potential of electronic health records. It is now possible to conduct some studies in weeks or months instead of years. The FDA mini-Sentinel system accesses 125 million patient records to generate several studies per week on drug safety questions [37]. Large biobanks are now coming on-line at Kaiser-Permanente, [38] the Veterans Health Administration [39], the ENCODE network, [40], and the UK Biobank [41]. Using these and other patient databases, researchers have been able to assess the unintended effects of treatments [42] and produce outcome findings comparable to RCTs [43].

Large future investments are now being considered that could offer extraordinary opportunities for researchers and a faster, more efficient infrastructure for rapid learning research. The NIH Director has proposed a new national patient-oriented research system with electronic health records databases, including genomics, for 20–30 million patients [44], and PCORI recently released a funding announcement to support development of the National Patient Centered Clinical Research Network [10]. The Big Data to Knowledge (BD2K) initiative [45] also provides the opportunity to leverage these large data sets for rapid research.

Researchers can accelerate the collective pace of learning with greater attention to reporting comparable data. There have been a number of efforts to encourage the use of common data elements [46]. Standardized outcome data are particularly problematic for patient-reported outcomes. To address this problem, there have been consensus measurement efforts such as PhenX [47] as well as efforts to co-calibrate various patient-reported outcome measures on a single metric [48].

The National Research Council report on *Precision Medicine* calls for a new science commons and national learning system that will revolutionize biomedical research, clinical care, and public health [49]. One of the benefits of such a system is that researchers can more readily target drug approval studies to predicted high-response populations, and cut years from the drug research process. Gleevec, an anti-cancer drug, was approved in a trial of only 54 patients because nearly all showed benefit [6]. With targeted therapeutics and research, it took only four years from target discovery to drug approval for the lung cancer drug Xalkori [50]. Rapid learning systems of large patient populations appear to provide the infrastructure to rapidly evaluate treatments.

## Conclusions

We have outlined a number of actions to enhance relevance, streamline design, speed review, and use new research infrastructures to make research more rapid, relevant, and responsive to the 21^st^ century demands on health research. This transformation to a rapid research learning system will require a concerted effort by research funders, academic institutions, healthcare systems, researchers, and a variety of practice, community, and policy stakeholders to produce a culture change among the health research community. Will we continue to use limited funds to support the currently slow and cumbersome research enterprise that produces costly results that may not be relevant or easily translated into practice, or are we willing to pursue alternative approaches that in other research disciplines have produced rapid and relevant improvements?

The rationale and opportunities for such a culture change have never been greater or rapid answers more needed. Our recommendations to speed research are undoubtedly incomplete, and we invite the research community to contribute additional recommendations to increase the speed and relevance of the research enterprise. This call to streamline and speed the research process is also likely to be met with skepticism, especially among those who fear that methodological rigor might be compromised in a quest for greater efficiency. We believe that the recommendations outlined in this paper can be achieved without compromising scientific rigor, and any efforts to streamline research should be judged based on methodological soundness. We are convinced, however, that the currently dominant health research paradigm is too slow and inefficient to address today’s challenges, and that we must produce a more rapid, responsive, and relevant research enterprise.

## Competing interests

The authors declare that they have no competing interests. Preparation of this manuscript was supported in part by a grant from The Robert Wood Johnson Foundation to George Washington University to the third author. The funders had no role in this manuscript. The opinions expressed are those of the authors and do not necessarily reflect those of the Robert Wood Johnson Foundation or the National Cancer Institute.

## Authors’ contributions

WTR led the writing and preparation of this paper. REG, LE, and APA all contributed sections to this paper, and revised and edited drafts of the paper. All authors read and approved the final manuscript.
